# CD155 immunoregulation as a target for natural killer cell immunotherapy in glioblastoma

**DOI:** 10.1186/s13045-020-00913-2

**Published:** 2020-06-12

**Authors:** Kyle B. Lupo, Sandro Matosevic

**Affiliations:** 1 Department of Industrial and Physical Pharmacy, Purdue University, West Lafayette, IN 47907 USA; 2 Purdue Center for Cancer Research, West Lafayette, IN 47906 USA

**Keywords:** Natural killer cells, Glioblastoma, CD155, TIGIT, Immunotherapy

## Abstract

Natural killer (NK) cells are powerful immune effectors, modulating their anti-tumor function through a balance activating and inhibitor ligands on their cell surface. Though still emerging, cancer immunotherapies utilizing NK cells are proving promising as a modality for the treatment of a number of solid tumors, including glioblastoma (GBM) and other gliomas, but are often limited due to complex immunosuppression associated with the GBM tumor microenvironment which includes overexpression of inhibitory receptors on GBM cells. CD155, or poliovirus receptor (PVR), has recently emerged as a pro-tumorigenic antigen, overexpressed on GBM and contributing to increased GBM migration and aggressiveness. CD155 has also been established as an immunomodulatory receptor, able to both activate NK cells through interactions with CD226 (DNAM-1) and CD96 and inhibit them through interaction with TIGIT. However, NK cell TIGIT expression has been shown to be upregulated in cancer, establishing CD155 as a predominantly inhibitory receptor within the context of GBM and other solid tumors, and rendering it of interest as a potential target for antigen-specific NK cell-based immunotherapy. This review will explore the function of CD155 within GBM as it relates to tumor migration and NK cell immunoregulation, as well as pre-clinical and clinical targeting of CD155/TIGIT and the potential that this pathway holds for the development of emerging NK cell-based immunotherapies.

## Introduction

Among the multiple elements contributing to the aggressive pathology of glioblastoma (GBM)—the most malignant brain tumor which currently stands with no curative treatment—is the emergence of CD155 as a pro-tumorigenic antigen [1–3]. A cell adhesion molecule of the immunoglobulin (Ig) superfamily, CD155 is a type I transmembrane glycoprotein that was first described as a poliovirus receptor (PVR) [4]. Though its expression can be detected at low levels on epithelial and endothelial cells in a variety of tissues, its overexpression on malignant cells has been associated with poor prognosis in patients with breast cancer [5], lung adenocarcinoma [6], pancreatic cancer [7], cholangiocarcinoma [8], melanoma [9], and various soft tissue tumors [10].

High-grade malignant gliomas, including GBM (grade IV), are associated with overexpression of CD155 [11], which was shown to contribute to cancer cell dispersal [1]. The receptor’s adhesive capacity has a well-established role in promoting migration and invasiveness of tumor cells [2]. Though CD155 has been shown to regulate certain immune cell responses such as graft-versus-host-disease [12], its role as a pro-tumorigenic antigen has received increased attention as of late. A dose-escalation trial of a recombinant nonpathogenic polio–rhinovirus chimera (PVSRIPO) delivered intratumorally to patients with grade IV glioma resulted in longer survival of treated patients at 24 and 36 months compared to patients treated historically [13].

CD155 exerts its functions by interacting with multiple ligands. Engagement of CD155 with ligands including CD226 (DNAM-1) and CD96 has been demonstrated to drive anti-tumor immune responses, particularly those by NK cells [14]. NK cells, moreover, express T cell immunoreceptor with Ig and ITIM domains (TIGIT), an immunoglobulin superfamily receptor, whose ligands include CD155, CD112, and CD113 [15]. TIGIT—which competes with DNAM-1 for binding to CD115—interacts with these receptors resulting in inhibition of NK cell anti-tumor function including impaired granule polarization and IFN-γ production [16, 17] and shows higher binding affinity for CD155 than CD112 [18]. Blockade of TIGIT on NK cells has resulted in restoration of powerful NK cell effector function in vivo and reversal of their functional exhaustion [19]. Partly because the expression of TIGIT is higher on NK cells compared to other lymphocytes [20], its role as an immune checkpoint within the CD155-TIGIT axis is receiving considerable attention [21, 22].

In GBM, TIGIT has been targeted in combination with PD-1 as a strategy to overcome adaptive resistance to single checkpoint blockade [23] while its overexpression on tumor-infiltrating immune cells correlates to their functional exhaustion [24]. Less is known about the prognostic significance of TIGIT in GBM, although evidence that it correlates negatively with patient survival, at least for low-grade glioma, has been suggested [23].

Despite demonstrated evidence that supports targeting the CD155-TIGIT axis as an immunotherapeutic strategy for solid tumors including GBM, the complexity of the pathway, the multiple related ligands, and receptors involved as well as its mobilization of immune responses by not just NK cells has caused many questions to remain open. Here, we present an evidence-based discussion on efforts aimed at understanding and exploiting CD155 as a target for immunotherapy of GBM mediated by NK cells.

## Expression and function of CD155 in GBM

CD155 is a cell surface receptor which belongs to the nectin and nectin-like family of immunoglobulin-like molecules that function as the receptor for poliovirus [4]. CD155 is overexpressed on GBM [1, 2] and other solid tumors, including melanoma [9], breast cancer [5], lung adenocarcinoma [6], pancreatic cancer [7], and a variety of soft tissue tumors [10]. In the context of GBM, Sloan et al. were among the first to describe the overexpression of CD155 in GBM using the U87-MG malignant glioma cell line and demonstrate that it plays a role in GBM invasiveness [2]. Upregulation of both membrane-bound and soluble CD155 in U87MG glioblastoma cells was subsequently reported by other groups [25]. Thompson et al. showed that a variety of low-grade and malignant pediatric brain tumors also overexpress CD155 and that targeting of CD155 on these tumors using an oncolytic virus could inhibit cellular proliferation of GBM [26]. Further confirming these findings have been large-scale IHC studies which have indicated the prevalence of CD155 in GBM and other gliomas [11, 27, 28].

The prognostic value of CD155 overexpression has been more extensively characterized for tumors other than GBM so far. Bevelacqua and colleagues measured overexpression of CD155 on melanoma cell lines WM35, A375, and M14 and samples from patients, and related such upregulation to increases in cancer cell migration and invasiveness [9]. Elsewhere, overexpression of CD155 was shown to correlate to a poorer prognostic outlook in patients with breast cancer [5], soft tissue sarcoma [10], lung adenocarcinoma, pancreatic cancer, malignant glioma, and colorectal carcinoma [2, 6, 7, 29]. This has been largely attributed to CD155’s demonstrated roles in the promotion of tumor invasion [1, 2, 30]. These findings have fueled interest in understanding the immunosuppressive role of CD155 in cancer via its interactions with its various ligands.

It has been established that CD155 and other nectin-like molecules, such as CD112 and CD113, play key roles in cell adhesion and migration [31]. CD155 interacts with a number of different ligands which are primarily present on immune cells, in particular cytotoxic T cells and NK cells. These interactions are both complex and varied and result in sometimes divergent immunoregulatory stimuli. CD155 ligands include inhibitory receptor TIGIT and activating receptors DNAM-1 and CD96 [32–34]. CD155 modulates the immunoregulation of T and NK cells through interactions with TIGIT, DNAM-1, and CD96, with, in particular, interactions between TIGIT and CD155 thought to result in severe immunosuppression of NK and cytotoxic T cells in the tumor microenvironment (TME) [35]. CD155 has also been shown to trans-interact with nectin-3, thus promoting cell migration through colocalization to epithelial cell junctions [36, 37]. In addition, CD155 overexpression has been linked to increases in cell proliferation, pitting CD155 as a key driver of cancer migration [2, 9, 11, 29].

In solid tumors, including gliomas, CD155 is recruited to the leading edge of the tumor where it co-localizes with actin and αv-integrin, compounds known to mediate cellular adhesion to the extracellular matrix (ECM) and to other cells [2, 38]. Following cellular adhesion, CD155 has been shown to increase activation of tyrosine kinases Src and FAK, which are recruited to the focal adhesions [2]. FAK is phosphorylated by Src, subsequently activating downstream molecules paxillin and p130Cas, which induce disassembly of focal adhesions, ultimately allowing for adhesion turnover and tumor migration [1]. Through this pathway, CD155 inhibits formation of mature focal adhesions and induces tumor cell migration in vitro and in primary brain tissues [1]. CD155 further mediates tumor progression by encouraging tumor cell growth through shortening of the G0/G1 phase of the cell cycle [3]. Heightened CD155 expression has additionally been associated with increase in VEGF expression and induction of angiogenesis, recognized markers of tumor invasiveness [7].

A number of groups have reported that CD155 also plays a key role in the invasiveness of GBM. Sloan and colleagues have shown that CD155 is upregulated in the GBM cell line U87MG, and knockdown of CD155 significantly inhibited chemoattractant-induced cell migration in transwell studies, suggesting CD155 has a role in tumor progression through metastasis and dispersal [2]. Upregulation of CD155 in GBM has also been shown to influence lymphocyte populations within the TME. Using surgical resection samples and matched blood from GBM donors, Lucca et al. showed that TIGIT^+^/DNAM-1^−^ tumor-infiltrating lymphocyte populations within GBM, which exhibited enhanced in vitro activation following TIGIT blockade, increased their presence from 25 to 60%, while such lymphocytes were typically absent within normal brain tissue [39]. While DNAM-1^+^ tumor-infiltrating lymphocytes were also observed at higher levels than in normal brain tissue, DNAM-1 was typically co-expressed with TIGIT, likely disrupting DNAM-1 homodimerization and rendering DNAM-1 nonfunctional. Further, the authors reported increases in peripheral TIGIT^+^/DNAM-1^−^ lymphocytes along with the localized increase of TIGIT^+^/DNAM-1^−^ lymphocytes within the TME, indicating that CD155 upregulation could affect expression of CD155 ligands on local and peripheral lymphocyte populations, though the exact mechanism has yet to be elucidated [39]. NK cell killing of neuroblastoma has also been shown to be mediated through CD155-DNAM-1 interactions, suggesting expression levels of CD155, TIGIT, and DNAM-1 play important roles in the regulation of activation and cytotoxic functions of NK cells in vivo, a concept which holds potentially significant clinical implications [40]. Collectively, these findings suggest that CD155/TIGIT appears to be a key interaction to consider when targeting immunomodulating pathways in GBM.

### NK immune regulation through CD155/TIGIT

**Fig. 1:**
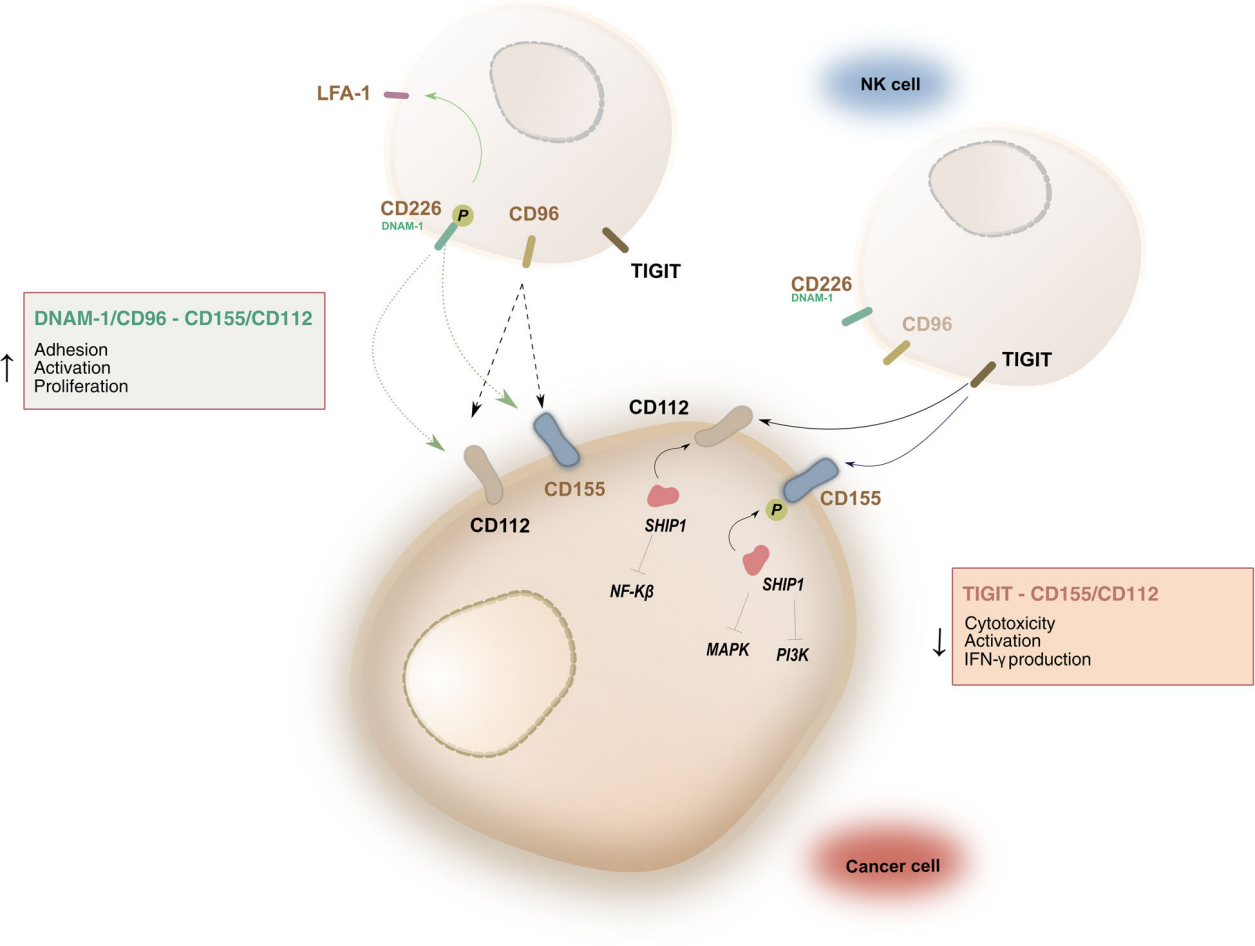
Diagram of CD155/CD112-mediated immunoregulatory pathway on NK cells. Interaction between TIGIT and CD155/CD112 induces inhibition of NK cell function via the phosphorylation of the ITIM domain and recruitment of SHIP1. This, in turn, inhibits MAPK, PI3K, and NF-κβ signaling. Interaction between DNAM-1/CD96 and CD155/CD112 provides an activating stimulus to NK cells. Upon binding, DNAM-1 relocates to lipid rafts where it homodimerizes and crosslinks with LFA-1 receptors. These cause phosphorylation of the Tyr^322^ residue of DNAM-1 resulting in NK cell cytolytic activity, cytokine secretion, and degranulation

In addition to cancer-driven immunometabolic reprogramming which impairs NK cell effector function in the solid tumor TME [41, 42], a number of immunosuppressive pathways in solid tumors that specifically affect NK cells involve various nectin-like molecules (NECL), such as CD155 (PVR) and CD112, which are expressed at low levels on normal cells but are highly upregulated in solid tumors, including GBM [2, 15]. CD155 and CD112 have been linked to tumor progression and migration in primary tumors and have also been shown to have an immunomodulatory role through interaction with DNAM-1, TIGIT, and CD96 on NK cells (Fig. 1) [2, 3, 43, 44]. While the link between upregulation of CD155 and tumor progression in GBM and other tumors has rendered CD155 a checkpoint target of potential interest, the related downregulation of DNAM-1 on NK cells in the TME within the context of CD155/TIGIT activity, coupled with its relatively lower affinity for CD155 and CD112 compared to that of TIGIT, limits NK cell activation and colors these pathways with additional complexity [40, 45].

Among the ligands that interact with CD155, TIGIT is of particular interest to the development of NK cell therapies [46] owing to its overexpression on NK cells in various cancers [20]. Due to its strong affinity for CD155, TIGIT is capable of interfering with positive DNAM-1 interactions and inducing NK cell inhibition. In addition, its role and function have been characterized extensively, making it a particularly intriguing target for immunotherapies. The TIGIT receptor on NK cells consists of an Ig-like extracellular domain along with a cytoplasmic domain consisting of an immunoreceptor tyrosine-based inhibitory motif (ITIM) and an immunoglobulin tail tyrosine (ITT) motif [18, 33, 47, 48]. TIGIT binds with high affinity to CD155, higher than that of DNAM-1, and to CD112, but with lower affinity, competitively inhibiting NK cell activation and functions [19, 49, 50]. While the exact intracellular mechanism is not completely understood, the ITIM domain of the TIGIT receptor plays a key role in NK cell inhibition, as it does in other inhibitory NK receptors (e.g., inhibitory KIRs, CD94/NKG2A) [44, 51, 52]. More specifically, following engagement of TIGIT with CD155, the ITIM domain of the TIGIT receptor is phosphorylated intracellularly, likely by a Src family kinase [53–55]. This is often achieved through crosslinking of an activating receptor to bring the Src kinase within close proximity of the ITIM domain [54]. ITIM phosphorylation then induces recruitment of SH2 receptors, SHIP-1, SHP-1, and SHP-2 [56]. Crosslinking of the ITIM domain with an activating receptor, often containing an ITAM domain, at cellular synapses allows these phosphatases to dephosphorylate molecules involved in NK cell activation, inhibiting NK cell cytolytic responses [56, 57]. Through this engagement of TIGIT with CD155 or CD112, NK cell cytolytic function is effectively diminished via reduction in IFN-γ secretion and granule polarization, ultimately interfering with the release of cytolytic granules by NK cells [16, 35]. Due to the high affinity with which TIGIT and CD155 bind, blockade of TIGIT has proven efficacious in enhancing NK cell immunotherapy [19].

Additionally, several other receptors expressed on NK cells interact with CD155 and CD112, including DNAM-1 and CD96. However, these receptors have been shown to have stimulatory functions in NK cells. DNAM-1 is an adhesion receptor containing two extracellular Ig-like domains attached to a cytoplasmic tail of three tyrosine residues. Unlike many other activating receptors, which induce activation through phosphorylation of intracellular ITAM domains, upon binding to CD155/CD112, homodimerization of DNAM-1 or crosslinking of DNAM-1 with other receptors activates NK cell cytolytic functions [32]. Specifically, activated DNAM-1 relocates to lipid rafts along the NK cell surface, binding with the actin cytoskeleton [58]. Here, the Ser^329^ residue located within the DNAM-1 intracellular domain is phosphorylated by protein kinase C (PKC) allowing for homodimerization of DNAM-1 molecules and crosslinking between DNAM-1 and LFA-1 receptors [59]. These interactions between DNAM-1 and LFA-1 have been shown to induce NK cell education [60, 61]. LFA-1 recruits Fyn Src kinase to phosphorylate the Tyr^322^ residue of DNAM-1, triggering phosphorylation of SLP76 and Vav1, and, subsequently, NK cell cytolysis, cytokine secretion, and degranulation via PLCg2 activation [60, 62, 63]. However, co-activation of other NK cell receptors is also required to prevent c-CbI inhibition of Vav1 and allow for DNAM-1-induced NK cell activation [64].

CD96, similarly to DNAM-1, has been implicated in the activation of human NK cell cytolytic functions [65]. While CD96 contains inhibitory intracellular ITIM domains, much like TIGIT, it also expresses a YXXM motif, conserved on many NK cell activating receptors, such as NKG2D, thus triggering activation upon phosphorylation [66]. This suggests that CD96 may play a regulatory role in NK cells, through either activation or inhibition in response to different cells, but this remains poorly understood. Nonetheless, CD96 has been shown to potentiate NK cell activation through cellular adhesion and interaction with CD155, but the exact mechanism and extent of activation induced by CD96 remains to be elucidated [65].

## Current immunotherapies targeting the CD155/TIGIT axis

With novel advances in activation strategies for NK cells, the development of new NK cell sources, as well as advancements in genetic engineering toward improved targeting approaches for various cancers [67–71], NK cell therapies have emerged as a potentially promising platform for treating difficult-to-cure solid tumors, including GBM [1, 2, 69, 72–75] and other gliomas [26]. Additionally, preclinical trials with combination therapies, utilizing donor-derived NK cells with checkpoint inhibitor antibodies and other molecules, have proven efficacious in reducing tumor-induced immunosuppression in GBM and enhancing NK cell responses [76, 77]. Apart from the clinical use of native, unmodified NK cells, therapies with engineered NK cells have been utilized in multiple clinical and preclinical studies to target a number of ligands which are overexpressed in GBM, including HER2 [78], IL-13Rα2 [79], EGFR [80], EGFRvIII [80], CSPG4 [77], and CD133 [81]. Despite emerging as a known immunosuppressor in GBM, however, CD155 has yet to be significantly targeted with NK cell therapies [72]. Burger et al. provide a thorough discussion of current NK cell therapies targeting important ligands on GBM and their implications for NK cell-based immunotherapies [72].

Nonetheless, together with the expanding body of work demonstrating CD155’s roles in tumor progression, evidence about its upregulation in GBM, and strong interaction with inhibitory TIGIT receptor on NK cells, targeting of the CD155/TIGIT axis is growing as a potentially powerful strategy to elicit substantial NK-mediated anti-tumor responses. However, with immunotherapies targeting CD155 and TIGIT within GBM being relatively new, clinical examples are limited. In fact, there are no active clinical trials utilizing NK or T cells to target CD155 on GBM, but there have been multiple studies targeting other ligands on GBM and malignant gliomas. Lee et al., for instance, observed that unmodified NK cells were able to exert significant cytolytic functions, including inhibition of GBM metastases, when adoptively transferred into a U87MG xenograft model in NSG mice [82]. Clinically, significant responses of autologous blood-derived NK cells to treat malignant gliomas have also been reported. In an early clinical study, NK cells were harvested from male and female patients and expanded using irradiated HFWT cells in RHAMα medium supplemented with autologous plasma and rhIL-2 for 21 days prior to infusion. Patients received one or more courses of treatment, consisting of weekly intra-tumoral and intravenous injections of NK cells for 3 weeks, and tumor responses were measured via MRI imaging. These treatments resulted in two of nine patients recording positive clinical responses, namely a reduction in tumor volume by more than 50%, which persisted for longer than 4 weeks [83]. In a current, separate phase 1 trial at M.D. Anderson Cancer Center, autologous, ex vivo-expanded, native NK cells are adoptively transferred into patients intravenously weekly for 1 to 3 weeks to target recurrent medulloblastoma and ependymoma and determine the side effects and maximum tolerated dose of this treatment (NCT02271711).

Adoptively-transferred NK cell therapies have also been combined with other therapeutics to improve NK cell anti-tumor effects in GBM. Wu et al. reported that stimulation with IFN-γ induced upregulation of NKG2D and NCR ligands on brain tumors, enhancing cell lysis by NK cells using surgical samples of two GBM tumors and two astrocytomas obtained from resected tumor tissue of human patients [84]. Elsewhere, blockade or depletion of immunosuppressive compounds, cytokines, and ligands has been shown to inhibit immunosuppression and enhance NK cell activation against GBM [85, 86]. Administration of recombinant antibodies targeting GD2 and inducing NK activation via IL-2 has also proven efficacious in treating neuroblastoma, further demonstrating that NK cell cytolytic functions can be enhanced in various ways to treat GBM and other brain tumors [87, 88].

There are also a number of active clinical trials targeting CD155 or TIGIT within GBM, albeit without NK cells. Results from a recent dose-escalation study treating patients with WHO grade IV malignant glioma through intra-tumoral delivery of the recombinant nonpathogenic polio–rhinovirus chimera (PVSRIPO) [13] showed promising anti-tumor responses. The study evaluated 7 doses of PVSRIPO in 61 patients and resulted in an increase in survival rate of 21% at 24 and 36 months when compared with historical controls (14% at 24 months and 4% at 36 months), with patients surviving for more than 70 months after infusion [13]. Several therapies have also been described that target the CD155/TIGIT axis in other tumor types, primarily utilizing checkpoint inhibitors and often targeting one or multiple other ligands in combination with CD155/TIGIT (Table 1).

**Table 1:** Currently active and ongoing clinical trials targeting the CD155/TIGIT axis

Immunotherapy type	Immunotherapy targets	Disease	Treatment	Phase	Clinical trial identifier	Sponsor
Checkpoint inhibitor	CD155 (PVR), PD-1	Solid tumors	COM701, Opdivo (nivolumab)	1	NCT03667716	Compugen Ltd, Bristol-Myers Squibb
Oncolytic virus	CD155 (PVR)	Melanoma	PVSRIPO	1	NCT03712358	Istari Oncology, Inc., Duke University
Oncolytic virus	CD155 (PVR)	Glioblastoma, malignant glioma	PVSRIPO	1	NCT01491893	Istari Oncology, Inc., National Cancer Institute (NCI), Brain Tumor Research Charity Grant, Duke University
Monoclonal antibody	TIGIT, PD-1	Metastatic solid tumors	BGB-A1217, tislelizumab	1	NCT04047862	BeiGene
Monoclonal antibody	TIGIT, PD-L1	Non-small cell lung cancer	Atezolizumab, MTIG7192A	2	NCT03563716	Genentech, Inc.
Monoclonal antibody	TIGIT, PD-L1	Small cell lung cancer	Tiragolumab atezolizumab, carboplatin, etoposide	3	NCT04256421	Hoffmann-La Roche
Monoclonal antibody	TIGIT, PD-L1	Non-small cell lung cancer	Atezolizumab, tiragolumab	3	NCT04294810	Hoffmann-La Roche
Monoclonal antibody	TIGIT, PD-1	Solid tumors	AB154, AB122	1	NCT03628677	Arcus Biosciences, Inc.
Monoclonal antibody	TIGIT, PD-1, A2aR/A2bR	Non-small cell lung cancer	Zimberelimab, AB154, AB928	2	NCT04262856	Arcus Biosciences, Inc.
Monoclonal antibody	TIGIT, D-L1, MEK1/2, VEGFR2, CXCR4, DPP-4	Gastric adenocarcinoma, esophageal carcinoma	5-Fluorouracil (5-FU), leucovorin, oxaliplatin, atezolizumab, cobimetinib, ramucirumab, paclitaxel, PEGPH20, BL-8040, linagliptin, cisplatin, tiragolumab	1/2	NCT03281369	Hoffmann-La Roche, Halozyme Therapeutics, BioLineRx, Ltd.

Inhibition of CD155 has also been shown to result in a reduction in tumor dispersion and invasiveness, as well as a reduction in secretion and activity of matrix metalloproteinase-2 (MMP-2) [89]. MMP-2 is a secreted endopeptidase which degrades ECM components and contributes to in vivo invasiveness of GBM [89]. Therapeutically, MMP-2 inhibition has been well established as contributing to the reduction of invasion of GBM cells [90, 91]. Therefore, CD155 inhibition alone can reduce production of MMP-2 and may be beneficial in slowing progression of GBM in vivo [2, 89]. However, clinical studies have so far mostly focused on inhibition of TIGIT, as CD155 interacts with other activation receptors on cells including NK cells (DNAM-1) and can positively stimulate immune responses in vivo. Inhibition of TIGIT strongly enhances NK cell function, including proliferation and production of pro-inflammatory cytokines [92]. Combinatorial checkpoint inhibition therapies targeting TIGIT and PD-1 on NK cells have also proven effective in the treatment of GBM [23]. Li et al. demonstrate that dual-targeting of the CD155/TIGIT and PD-1/PD-L1 axes in NK and T cells improved effector cell-mediated antitumor immunity in solid tumors [93]. In their study, triple blockade of PD-1, TIGIT, and CD96 significantly inhibited tumor growth in a B16F10 melanoma BALB/c WT mouse model [93]. Separately, Zhang et al. reported that TIGIT blockade could reverse TME-induced exhaustion of NK cells and restore NK cell anti-tumor functions [19]. Their study measured functional exhaustion of NK cells, extracted from tumor-bearing mice, through the expression of IFN-γ, TNF, CD107a, and cytotoxic molecule TRAIL, noting a reduction in expression of these markers which was restored through TIGIT blockade. Elsewhere, Johnston et al. reported that TIGIT blockade alone could enhance effector function of CD8+ T cells after adoptive transfer but did not measure this effect in NK cells [94].

## Engineering NK cells to overcome TIGIT-induced inhibition

The immunomodulatory role of CD155/CD112, through its interaction with both DNAM-1 and TIGIT on NK cells and its heightened expression on GBM, represent an opportunity for advances in immunotherapy treatments. By targeting this axis in NK cells, through engineered cell therapies and combinatorial antibody/cell therapy approaches, it is possible to suppress CD155-induced inhibition and enhance the natural cytolytic functions of NK cells. Recent advances in chimeric antigen receptor (CAR)-NK therapies have allowed engineered NK cells to find their way to the clinic [95], where they have predominantly targeted CD19 on B cell malignancies [74, 96, 97], but also a growing number of solid tumor ligands, such as HER2 and MUC1 [98–100].

Engineered NK cells have also been under active investigation as immunotherapeutic effectors against GBM [80, 101]. Indeed, several preclinical studies utilizing CAR-engineered NK cells have targeted ligands on GBM, although they have not specifically targeted TIGIT/CD155 induced immunosuppression. A phase 1 clinical trial aimed at treating GBM patients with CAR-NK-92 cells engineered with a second-generation CAR targeting HER2 (NCT03383978) [102] is currently underway at Johann Wolfgang Goethe University Hospital. In this dose-escalation study, between 1 × 10^7^ and 1 × 10^8^ NK-92 cells have been adoptively transferred into treated subjects, with the aim of identifying optimal dose ranges for single and repeated injections.

Mueller et al. engineered an NK cell line (YTS) to express a CAR targeting EGFRvIII on glioblastoma, as well as a CXCR4 chemokine receptor [101]. While NK cells expressing EGFRvIII-CARs alone effectively delayed tumor growth, the combined EGFRvIII/CXCR4 targeted therapies significantly increased survival in mice with U87MG xenografts over EGFRvIII-CAR NK cells or controls [101]. Han et al. engineered a variety of NK cells (cell lines NK-92 and NKL, and primary NK cells) with CARs targeting EGFR and EGFRvIII and noted an enhanced cytolytic ability and cytokine secretion of these engineered NK cells in response GBM cells [80]. Elsewhere, enhanced cytolytic function of cetuximab-based CAR-engineered NK-92 cells, targeting both EGFR and EGFRvIII on GBM with a single ligand, has been reported. The dual antigen-targeting approach was used to prevent immune escape, a feature previously described for GBM [103]. Other ligands expressed highly on GBM have also been investigated through CAR-NK therapies, including Erb2 and HER2 [78]. While no studies have been reported targeting the CD155/TIGIT axis with engineered NK cells, the emerging role CD155 in GBM progression and immunoregulation makes it an increasingly attractive target for this space.

## Clinical outlook and future directions

It is becoming evident that targeting the TIGIT/CD155 axis through cellular engineering or with antibodies holds tremendous potential to induce significant anti-GBM responses. The demonstrated role of this axis in regulating NK cell immune responses makes NK cells an attractive immunotherapeutic proposition in GBM immunotherapy via this pathway. NK cell-based therapies alone have proven effective in treating a variety of cancers [5–7, 9, 10, 104], and recent targeting of GBM with NK cell therapies has proven promising [78, 80, 101, 103]. CD155 represents a potentially significant target with implications both in the regulation of immune cells and tumor progression, proliferation, and invasion [89, 92]. Though there is great complexity, including functional divergence, of the many pathways and ligands involved in the various CD155 signaling interactions, clinical trials targeting CD155 within GBM have already proven efficacious, with intratumoral delivery of a recombinant nonpathogenic polio-rhinovirus chimera (PVSRIPO) yielding a 3-year survival rate of 21% as compared to historical 3-year survival rates of 4% [13]. Therefore, utilizing NK cell therapies to target the CD155/TIGIT axis can be exploited as a strategy for treating GBM toward improved clinical outcomes. The use of combinatorial therapies targeting CD155 together with other ligands overexpressed in the GBM TME will likely yield the most robust and durable clinical responses. This is already evident by the fact that most of the current clinical therapies targeting CD155 also target other checkpoints (PD-1/PDL-1, CXCR4, etc.) present within solid tumors. Ultimately, the complexity of the TME of solid cancers, particularly that of GBM, is likely to require concomitant targeting of multiple ligands and cytokines for therapeutic effect and a better understanding of how the various interactions CD155 functions along guide effector functions of NK cells.

## Data Availability

Data sharing is not applicable to this article as no datasets were generated or analyzed during the current study.
